# Glacial Steady State Topography Controlled by the Coupled Influence of Tectonics and Climate

**DOI:** 10.1029/2017JF004559

**Published:** 2018-06-19

**Authors:** Günther Prasicek, Frédéric Herman, Jörg Robl, Jean Braun

**Affiliations:** ^1^ Institute of Earth Surface Dynamics University of Lausanne Lausanne Switzerland; ^2^ Department of Geography and Geology University of Salzburg Salzburg Austria; ^3^ GFZ German Research Centre for Geosciences Helmholtz Centre Potsdam Potsdam Germany; ^4^ Institute of Earth and Environmental Science University of Potsdam Potsdam Germany

**Keywords:** glacial equilibrium, steady state topography, glacial erosion, glacial buzzsaw, rock uplift‐relief scaling, scaling relation

## Abstract

Glaciers and rivers are the main agents of mountain erosion. While in the fluvial realm empirical relationships and their mathematical description, such as the stream power law, improved the understanding of fundamental controls on landscape evolution, simple constraints on glacial topography and governing scaling relations are widely lacking. We present a steady state solution for longitudinal profiles along eroding glaciers in a coupled system that includes tectonics and climate. We combined the shallow ice approximation and a glacial erosion rule to calculate ice surface and bed topography from prescribed glacier mass balance gradient and rock uplift rate. Our approach is inspired by the classic application of the stream power law for describing a fluvial steady state but with the striking difference that, in the glacial realm, glacier mass balance is added as an altitude‐dependent variable. From our analyses we find that ice surface slope and glacial relief scale with uplift rate with scaling exponents indicating that glacial relief is less sensitive to uplift rate than relief in most fluvial landscapes. Basic scaling relations controlled by either basal sliding or internal deformation follow a power law with the exponent depending on the exponents for the glacial erosion rule and Glen's flow law. In a mixed scenario of sliding and deformation, complicated scaling relations with variable exponents emerge. Furthermore, a cutoff in glacier mass balance or cold ice in high elevations can lead to substantially larger scaling exponents which may provide an explanation for high relief in high latitudes.

## Key Points

- Glacial steady state topography is predicted based on the shallow ice approximation and a glacial erosion rule
- Ice thickness and surface slope in glacial topographic steady state depend on rock uplift rate and mass balance
- Compared to the fluvial realm, altitude‐dependent mass balance may reduce sensitivity of glacial landscapes to rock uplift rates

## 1 Introduction

Glaciers shape major mountain landscapes worldwide and scour characteristic landforms such as cirques and overdeepened troughs (e.g., Davis, 1906; Ehlers & Gibbard, 2007; Penck, 1905). The resulting landscapes are clear evidence for a climate control on the shape of the Earth's surface (e.g., Davis, 1906; Herman et al., 2013; Hinderer, 2001; Peizhen et al., 2001; Penck, 1905; Robl et al., 2015). Glaciers are thought to increase local relief below the equilibrium line altitude (ELA) through the carving of deep troughs by channeled ice, while they reduce relief above the ELA due to distributed glacial erosion (e.g., Brardinoni & Hassan, 2006; Brocklehurst & Whipple, 2004; Egholm et al., 2017; Steer et al., 2012). Over long timescales this may lead to a hypsometry that displays a maximum at the ELA due to the altitude dependence of glacier mass balance and consequently ice flux and glacial erosion, a mechanism known as the glacial buzzsaw. The buzzsaw effect, however, is only loosely defined, and some interpretations include a governing influence of rock uplift rate or total rock column uplift on glacial topography (Brocklehurst & Whipple, 2007; Pedersen et al., 2010), while others claim a purely climatic control on mountain height through glacial processes (Brozović et al., 1997; Mitchell & Montgomery, 2006). Furthermore, the time frame of the buzzsaw effect has not been clearly articulated. It may describe the climate‐induced rapid destruction of fluvial relief in an overall transient system state but also a glacial steady state scenario where climate controls how rock uplift and erosion rates can balance each other. Empirical studies have found evidence for both an increase (e.g., Champagnac et al., 2014; Montgomery, 2002; Shuster et al., 2011; Valla et al., 2011) and decrease (e.g., Schlunegger & Hinderer, 2003; Tomkin & Braun, 2002) of relief due to glacial erosion, and a concentration of peak height and hypsometric maxima within a certain vertical distance to the Pleistocene mean ELA (e.g., Egholm et al., 2009), as well as relief being similar in glacial and fluvial terrain (Brocklehurst & Whipple, 2002) and invariant over glacial‐interglacial cycles in regions of very high uplift (Herman et al., 2010). Furthermore, headward propagation of glacial erosion (Shuster et al., 2011), a dependence of glacial erosion on valley shape (Leith et al., 2014; Pedersen & Egholm, 2013), and a control of rock uplift rates on the fluvial overprinting of glacial topography (Prasicek et al., 2015) have been proposed. It remains difficult to put each of these findings into a theoretical context of glacial landscape evolution as long as the characteristics and dependencies of a glacial steady state topography have not been further constrained.

The physics of glaciers has been investigated for decades, and theoretical models of different complexity exist to describe the flow of ice (e.g., Gagliardini et al., 2013; Hutter & Hughes, 1984; Nye, 1952). Furthermore, our understanding of glacial erosion has significantly improved in recent years due to a wealth of new field data (Herman et al., 2015; Koppes & Montgomery, 2009; Koppes et al., 2015) and improved mathematical descriptions (e.g., Anderson et al., 2006; Braun et al., 1999; Egholm et al., 2011; Hallet, 1979, 1996; Harbor, 1992; MacGregor et al., 2000). Similarly, the long‐term impact of fluvial erosion on mountain landscapes and its relation to tectonic and climatic forcing are well constrained (e.g., Goren, 2016; Kirby & Whipple, 2012; Lague, 2014; Perron & Royden, 2013; Robl et al., 2017; Whipple & Tucker, 1999; Whittaker, 2012) and the first‐order applicability of simple erosion models to describe fluvial landscape evolution has been tested in numerous experiments (e.g., Montgomery, 2001; Snyder et al., 2000; Wobus et al., 2006). However, a profound understanding of the impact of tectonics and climatic preconditioning on fluvial landscapes is based on predicting steady state end‐members and how fluvial topography will evolve under given conditions (e.g., Whipple & Tucker, 1999). In spite of that, a glacial counterpart to this approach has not yet been fully developed and a proper description of glacial steady state topography is lacking.

This may in part reflect the different disciplines involved. Physicists and glaciologists have developed sophisticated ice sheet models, but their research focuses rather on the physics of ice flow and the relation between glaciers, climate, and environmental change than on landscape evolution over geological timescales. In addition, glaciers have occupied most mountain landscapes only since the last 2 Myr and under strongly varying climatic conditions (e.g., Ehlers & Gibbard, 2007; Lisiecki & Raymo, 2007; Sosdian & Rosenthal, 2009), a time span possibly too short and climatically too unstable for most high‐mountain landscapes to reach or even closely approach a glacial steady state. However, theoretical constraints on the direction of landscape evolution and its governing parameters are critically needed to improve our understanding of the entire system and serve as a benchmark against which other system states can be tested.

In this study, we derive constraints on glacial steady state longitudinal profiles from the shallow ice approximation (SIA) and a glacial erosion rule. Our approach extends the work of Headley et al. (2012) by using a different method to solve the SIA at topographic equilibrium and, more importantly, by treating glacial landscapes as a coupled system in which glacial topography is the product of the different interactions between tectonic and climatic processes. This allows us to investigate the potential links and feedbacks between rock uplift rate and glacier mass balance. While rock uplift rate produces glacial relief, ice flux depending on glacier mass balance tends to reduce it above the ELA. We compare glacial steady state topography to its fluvial equivalent and highlight the altitude‐dependent glacier mass balance as an independent variable that reduces the sensitivity of glacial topography on rock uplift rate. Our theoretical results help to put constraints on the nature of the glacial buzzsaw effect and to explain the observed dependencies of glacial relief on rock uplift rates, as well as the presence of high topography in high latitudes.

## 2 Shallow Ice Solution for Glacial Equilibrium Profiles

### 2.1 Analytical Solution

Here we first follow the idea of Headley et al. (2012) to derive an analytical solution to the equations governing a glacier steady state longitudinal profile using the SIA and an erosion rule but propose a new method that leads to a more versatile solution. Subsequently, we expand our analyses beyond the analytical approach and treat glacial landscapes as a coupled system in which glacial topography is a product of the interaction between tectonics and climate.

The change of bedrock elevation over time in an active orogen is commonly described as the result of a competition between rock uplift rate *U* and erosion rate ė:

(1) $$\frac{\mathrm{\partial z}}{\mathrm{\partial t}}=U-ė$$

Glacial erosion ė is commonly modeled as proportional to basal sliding velocity *u*_*s*_ (Hallet, 1979; Hallet et al., 1996):

(2) $$ė=K_{g}|u_{s}{|}^{l}$$

where *K*_*g*_ (m^1 − *l*^ year^*l* − 1^) is an erodibility coefficient (or erodibility) and *l* an exponent likely to lie between 1 and 2 (e.g., Harbor, 1992; MacGregor et al., 2009; Tomkin & Braun, 2002). Assuming topographic steady state, the rate of change of topography is zero and *U* must equal ė at all points under the glacier. Consequently, *u*_*s*_ is given by

(3) $$u_{s}={\left(\frac{U}{K_{g}}\right)}^{\frac{1}{l}}$$

If rock uplift rate and erodibility are uniform under the glacier, erosion rate and hence sliding velocity must be uniform as well (equations (2) and (3)). The exponent *l* is only needed to calculate *u*_*s*_ in equation (3) and can thus be chosen with no influence on the subsequent calculation procedure.

To determine the shape of the glacier, a glacier mass balance has to be prescribed. Here the glacier mass balance is assumed to be linearly dependent on the position along the glacier (*x*, measured in a downstream direction) via the mass balance gradient *β*_*x*_ and in relation to the horizontal position of the equilibrium line, *E*_*x*_. This is consistent with the approach of Headley et al. (2012). The mass balance can then be integrated from the summit of the glacier to estimate ice flux *q* at any point *x* along the glacier:

(4) $$q=\int_{0}^{x}-\beta_{x}\left(x-E_{x}\right)\;dx$$

If we further assume that the glacier is in equilibrium with climate such that, at every point along the glacier, mass outflux exactly balances mass influx plus (or minus) the ice accumulation (or ablation), *q* can also be written as a function of mean deformation velocity (*u*_*d*_), sliding velocity (*u*_*s*_), and ice thickness (*H*):

(5) $$q=(u_{d}+u_{s})H$$

Under the SIA, both *u*_*d*_ and *u*_*s*_ can be written as a function of deformation (*f*_*d*_) and sliding (*f*_*s*_) parameters, *H* and ice surface slope d*h*/d*x* (equations (6) and (7)), where *n* = 3 is Glen's flow law exponent for ice (Cuffey & Paterson, 2010).

(6) $$u_{d}=f_{d}H^{\left(n+1\right)}{\left(\frac{dh}{dx}\right)}^{n}$$

(7) $$u_{s}=f_{s}H^{\left(n-1\right)}{\left(\frac{dh}{dx}\right)}^{n}$$

The deformation and sliding parameters are themselves functions of the ice deforming and sliding factors, *A*_*d*_ and *A*_*s*_, respectively, according to

(8) $$f_{d}=3.16\times 10^{7}{\left(\mathrm{\rho g}\right)}^{n}A_{d};f_{s}=3.16\times 10^{7}{\left(\mathrm{\rho g}\right)}^{n}A_{s}$$

where *ρ* is ice density and *g* gravitational acceleration.

By dividing equation (6) by equation (7), *u*_*d*_ can be expressed in terms of *u*_*s*_. Equation (5) can then be rewritten as

(9) $$q=\frac{f_{d}u_{s}}{f_{s}}H^{3}+u_{s}H$$

Equation (9) is a cubic equation that has only one real solution for *H*. From this, *H* can be found analytically at every point along the glacier length where the ice flux is known (i.e., according to equation (4). Equation (7) can now be solved for ice surface slope d*h*/d*x*, which remains as the only unknown. The ice surface elevation (*h*) of a steady state glacier longitudinal profile can finally be obtained by integrating ice surface slope and considering the ELA, *E*.

(10) $$h=E+{\left(\frac{u_{s}}{f_{s}}\right)}^{\frac{1}{n}}\int_{0}^{L}\;H^{\frac{1-n}{n}}\;dx$$

**Figure 1:**
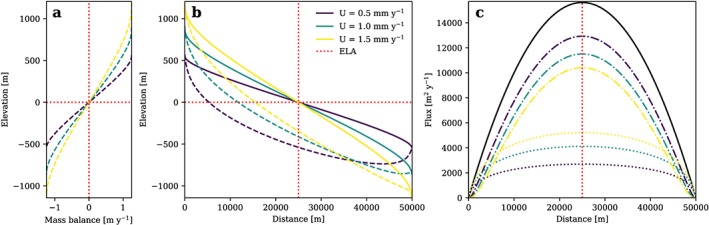
(a) Glacier mass balance, (b) ice surface (solid line) and bedrock surface (dashed line), and (c) total flux (solid black line), flux from deformation (dash‐dotted lines), and flux from sliding (dotted lines) of a glacier longitudinal profile in topographic steady state plotted for l = 1 and different rock uplift rates. Glacier mass balance defined to depend linearly on x. Note that the flux components of sliding and deformation adjust due to variations of surface slope and ice thickness with U, while total flux is a function of x and hence remains unchanged in this scenario. ELA = equilibrium line altitude.

Bedrock topography *z* can further be obtained by subtracting *H* from *h*. This approach produces a glacier with an ice flux that is symmetric about the ELA (Figure 1), here assumed to be at a reference elevation 0. All parameters used to produce this and all following model results are listed in Table 1.

**Table 1:** Constants Used to Model Glacial Steady State Topography in This Study

Parameter	Description	Value	Unit	Source
$A_{d_{E}}$	Ice deformation factor at ELA	2.4 × 10^−24^	Pa^−3^ · s^−1^	Cuffey and Paterson (2010)
$A_{s_{E}}$	Ice sliding factor at ELA	1.7 × 10^−19^	Pa^−3^ · m^2^ · s^−1^	Headley et al. (2012)
*β*	Glacier mass balance gradient over *z*	1 × 10^−3^	—	Hagen et al. (2003)
*β*_*x*_	Glacier mass balance gradient over *x*	5 × 10^−5^	—	—
$B_{\max}$	Maximum glacier mass balance	0.4	m/year	Oerlemans and Hoogendoorn (1989)
*δ*	Temperature lapse rate with *z*	6 × 10^−3^	K/m	Rolland (2003)
*E*	ELA	0	m	—
*E*_*x*_	Horizontal position of equilibrium line	2.5 × 10^4^	m	—
*g*	Gravitational acceleration	9.81	m/s^2^	—
*γ*	Glacier mass balance ratio	1.5	—	—
*k*	Valley width shape parameter	1.4 × 10^−4^	—	Oerlemans (2008)
*K*_*f*_	Erodibility in fluvial erosion rule	1 × 10^−4^	—	Wobus et al. (2006)
*K*_*g*_ (*l*=1)	Erodibility in glacial erosion rule	1 × 10^−4^	—	Humphrey and Raymond (1994)
*K*_*g*_ (*l*=2)	Erodibility in glacial erosion rule	1 × 10^−5^	m^−1^ year	Herman et al. (2015)
*l*	Exponent of glacial erosion law	1; 2	—	Herman et al. (2015)
*n*	Exponent from Glen's law	3	—	Cuffey and Paterson (2010)
*ρ*	Ice density	910	kg/m^3^	—
*w*_0_	Valley width at terminus	500	m	—
*ζ*_*d*_	Temperature‐deformation factor	2 × 10^−3^	—	Cuffey and Paterson (2010)
*ζ*_*s*_	Temperature‐sliding factor	9 × 10^−3^	—	—

*Note.* Glacier mass balance gradients chosen to mimic empirical data of mountain glaciers in humid areas (e.g., Cuffey & Paterson, 2010; Fischer, 2010; Meier & Post, 1962). ELA = equilibrium line altitude.

### 2.2 Glacier Mass Balance as a Function of Elevation

In contrast to analytical solutions for fluvial erosion based on the widely used stream power law (Flint, 1974; Whipple & Tucker, 1999), the glacier mass balance and the ELA used to model glacial landscapes depend strongly on temperature and hence altitude. A mass balance prescribed via *x* (equation (4)) inherently contains information on ice surface elevation as ice surface slope depends on ice thickness and hence flux (equation (10)). Unfortunately, the dependence of the mass balance on *x* and the constraints it imposes on the ice surface in a glacial steady state are a priori unknown. This is depicted in Figure 1 where glacial steady state longitudinal profiles are modeled under the assumption of a linear variation of glacier mass balance with *x*. The linear dependence of mass balance on *x* leads to a curved mass balance over elevation (Figure 1a). This indicates a mass balance gradient that constantly and systematically changes with altitude while a constant mass balance gradient, that is, a linear relation of mass balance with altitude, at least to a certain cutoff, is suggested by empirical data (e.g., Benn & Lehmkuhl, 2000; Hagen et al., 2003; Oerlemans & Hoogendoorn, 1989). Furthermore, the mass balance gradient in the analytical solution varies with rock uplift rate (Figures 1a and 1b), while the total flux remains unaffected (Figure 1c, black line). This contradicts the nature of the glacier mass balance gradient as a climatic variable which depends on temperature and precipitation and should thus be independent from tectonic influences, at least as long as orographic effects are neglected. To overcome these issues, we extend our study beyond the work of Headley et al. (2012) and consider glacier mass balance as a function of altitude instead of distance along the glacier. For this, it is convenient to introduce a glacier mass balance gradient over elevation, *β* = d*B*/d*z*, where *B* is the glacier mass balance. In this way an empirically determined relation between glacier mass balance and altitude can be used to derive glacial steady state topography. Consequently, the SIA solution for topographic equilibrium must involve two sets of independent variables, one in the horizontal domain including rock uplift rate and erodibility and one in the vertical domain including glacier mass balance.

To model glacial equilibrium topography in a coupled system with *U* and *K*_*g*_ prescribed as independent variables in the horizontal and glacier mass balance gradient *β* in the vertical domain, we follow an iterative approach. This is necessary, because steady state ice surface elevation is a priori unknown but needed to calculate glacier mass balance *B* from an empirical *β*. We thus prescribe the glacier mass balance as a function of *x* (position along the glacier) only in the initial model iteration to arrive at a first guess for ice surface elevation. For this, glacier mass balance is initially assumed to vary linearly with *x* as defined by an arbitrary mass balance gradient *β*_*x*_. The elevation of the ice surface *h* predicted in the initial run is then used to calculate an updated glacier mass balance *B* from

**Figure 2:**
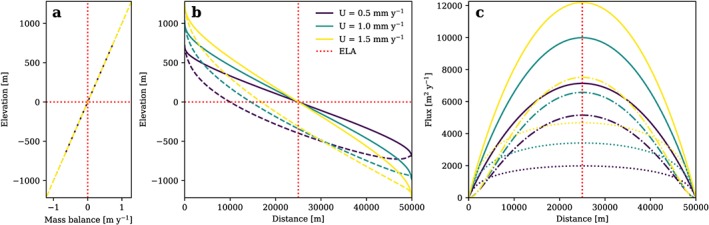
(a) Glacier mass balance, (b) ice surface (solid line) and bedrock surface (dashed line), and (c) total flux (solid lines), flux from deformation (dash‐dotted lines), and flux from sliding (dotted lines) of a glacier longitudinal profile in topographic steady state plotted for l = 1 and different rock uplift rates. Mass balance defined to depend linearly on z. Note that the mass balance gradient is constant over elevation and for all U while all flux components change with relief. ELA = equilibrium line altitude.

(11) B=β(h−E)

where *E* is the ELA and with *β* taken from observations (Table 1) and assumed to be constant in this first simple formulation. *B* is then used to update the ice flux, which is fed back into equation (9) to correct ice thickness and subsequently ice surface elevation (equation (10)). The procedure is repeated until the difference between two successive estimates of the glacier mass balance falls below a defined convergence criterion. In this way we obtain glacier mass balance, ice flux, and ice thickness along an equilibrium glacier, as well as the corresponding ice surface and bed topographies (Figure 2). *β* is now independent of *U* and *K*_*g*_ (Figure 2a), while ice flux changes due to the dependence of ice surface elevation on *U* and *K*_*g*_ (Figure 2c). In this simple example glacier mass balance depends linearly on elevation, which again makes the ice flux symmetric about the ELA. Consequently, maximum ice thickness and flux occur at the ELA. This glacial steady state scenario of a simple temperate glacier with *n* = 3, *U* = 1×10^−3^ m/year, *K*_*g*_=1 × 10^−4^, and *l* = 1 will be used as a reference when analyzing how model parameters and assumptions influence the predicted glacier geometry. Our reference choice of *n* is based on a review of field studies on glacier dynamics by Cuffey and Paterson (2010). The reference *U* represents a rock uplift rate magnitude that has been observed in many mountain ranges. *K*_*g*_=1 × 10^−4^ together with *l* = 1 in the glacial erosion rule have been suggested in several studies (Humphrey & Raymond, 1994; Koppes & Montgomery, 2009; Riihimaki et al., 2005) and are used in glacial erosion models (e.g., Harbor, 1992; Headley et al., 2012; Herman & Braun, 2008; although other values have been suggested recently; see section 3). Note that *E* and the horizontal position of the equilibrium line (*E*_*x*_, equation (4)) are predefined and an *E* ≠ 0 has to be considered in equation (10). We assume *E*_*x*_ to be constant and independent of *U* in our model runs.

#### 2.2.1 Nonuniform Glacier Mass Balance Gradients

Glacier mass balance depends on altitude through a broad range of processes: the atmospheric lapse rate (i.e., the temperature gradient over elevation), which, for a given total amount of precipitation, determines the proportion falling as snow, the ice and snow ablation, the short‐wave radiation absorption, and the incoming long‐wave radiation (e.g., Benn & Lehmkuhl, 2000; Oerlemans & Hoogendoorn, 1989; Réveillet et al., 2017). For contiguous, snowfall‐fed, clean‐ice glaciers these dependencies result in an approximately linear glacier mass balance over elevation which is simulated in the approach described above (equation (11)). This does not take into account that avalanching, debris cover, and topographic effects can have an influence on patterns of accumulation and ablation in high‐mountain environments (e.g., Anderson, 2000; Benn & Lehmkuhl, 2000). Furthermore, both mountain glaciers and ice shields have been found to show a glacier mass balance cutoff due to the depletion of moisture available for precipitation at higher elevations (e.g., Huss et al., 2008; Kessler et al., 2006).

**Figure 3:**
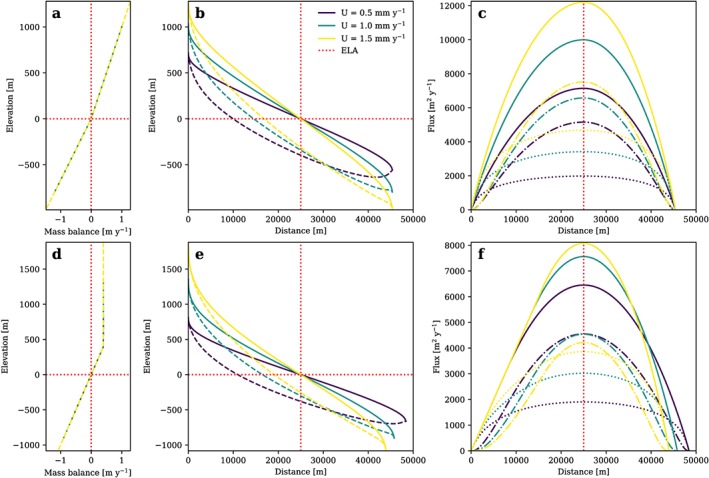
(a) Glacier mass balance, (b) ice surface (solid line) and bedrock surface (dashed line), and (c) total flux (solid lines), flux from deformation (dash‐dotted lines), and flux from sliding (dotted lines) of a glacier longitudinal profile in topographic steady state plotted for l = 1, different rock uplift rates, and a mass balance ratio γ of 1.5. (d–f) Similar plots for a mass balance cutoff of 0.4 m/year. ELA = equilibrium line altitude.

In our glacial equilibrium approach any variation of glacier mass balance over elevation can be prescribed. We introduce two simple scenarios in addition to the case of a linear mass balance with elevation. First, mass balance gradients may differ upstream and downstream of a certain point along the glacier, here assumed to be the ELA, as defined by a glacier mass balance ratio *γ*

(12) $$B=\left\{\begin{array}{cc} h>E, & \beta (h-E)\\ h<E, & \beta \gamma (h-E) \end{array}\right.$$

which can represent, for example, dirty ice (*γ* > 1) or a thick debris cover (*γ* < 1) below the ELA (e.g., Benn & Lehmkuhl, 2000). Example profiles for different *U* are plotted in Figures 3a–3c and show that, in cases where *γ* ≠ 1, the symmetry of the ice flux about the ELA is lost. In the particular case shown in Figures 3a–3c, the part of the glacier below the ELA has shortened due to the increase in mass balance gradient (*γ* > 1). From the ELA to the terminus the flux decreases more rapidly than it increases from the top to the ELA.

Second, we consider the existence of a mass balance cutoff, *B*_*m*_, such that mass balance is uniform above a certain elevation, according to

(13) $$B=\min\left\{\beta (h-E),B_{m}\right\}$$

In Figures 3d–3f *B*_*m*_ is set to 0.4 m/year and is reached 400 m above the ELA. This leads to a reduction in glacier mass balance and flux, and, consequently, to lower ice thickness, steeper ice surface slopes, an increase in glacial steady state relief, and a decrease in glacier length. In both cases of mass balance change, maximum *H*, and *q* are still located at the ELA.

### 2.3 Ice Temperature

The influence of a vertical atmospheric temperature gradient on ice temperature and hence ice deformation and sliding is not included in the formulation of the SIA (Hutter & Hughes, 1984). While heat conduction in ice is physically well constrained (e.g., Bird et al., 2007) and the relationship between ice temperature and the deformation factor *A*_*d*_ has been experimentally determined (Cuffey & Paterson, 2010), the ice sliding law is poorly known (Schoof, 2005) and the influence of ice temperature on sliding is difficult to directly measure in the field. This is because there exists only a very limited number of glaciers where access to their base is possible and also because the relationship between temperature and sliding is influenced by a number of factors that are difficult to constrain and differentiate, such as basal water pressure and thickness and solute content of the basal water film (e.g., Bartholomaus et al., 2008; Shreve, 1984). Despite this, a first‐order approximation of the effect of ice temperature on sliding may be obtained by prescribing an air temperature gradient with elevation, *δ*, equating ice temperature with air temperature above the ELA, and assuming sliding and deformation parameters for ice just below the melting point below the ELA. Furthermore, the presence of some amount of basal sliding even at temperatures below the pressure melting point (e.g., Cuffey et al., 1999; Echelmeyer & Zhongxiang, 1987; Shreve, 1984) has to be assumed to be able to solve for steady state topography. In case of a glacier frozen to its bed and thus zero sliding, there is no erosion to balance rock uplift and thus no steady state solution can be found.

Above the ELA, the change of *A*_*d*_ with temperature can be described by the following power law:

(14) $$A_{d}=A_{d_{E}}^{1-\mathrm{h\delta}\zeta_{d}}$$

as derived experimentally (Cuffey & Paterson, 2010). $A_{d_{E}}$ is the deformation factor at the ELA where ice temperature is assumed to be just below the melting point, and *ζ*_*d*_ is scaled to fit the decrease of ice deformation with temperature to the experimental data. We describe the change of *A*_*s*_ with elevation by a similar function but assume a faster decrease with decreasing temperature. In this way, glacier motion asymptotically approaches zero with decreasing temperatures (and thus increasing elevation) and deformation becomes more and more dominant, a behavior that can be expected in glaciers. The deformation and sliding parameters are then obtained from

(15) $$f_{d}=3.16\times 10^{7}{\left(\mathrm{\rho g}\right)}^{n}A_{d_{E}}^{1-\mathrm{h\delta}\zeta_{d}};f_{s}=3.16\times 10^{7}{\left(\mathrm{\rho g}\right)}^{n}A_{s_{E}}^{1-\mathrm{h\delta}\zeta_{s}}$$

as explained previously (equation (8)). Note that this formulation is only a very simple surrogate for both the temperature of glacier ice and the resulting differences of glacier motion with elevation. In fact, glaciers only reach down below the ELA because ice temperature does not equal air temperature, a result of conductive and advective heat transfer (e.g., Cuffey & Paterson, 2010). However, this approach predicts the first‐order influence of temperature change with elevation above the ELA on glacial steady state topography.

**Figure 4:**
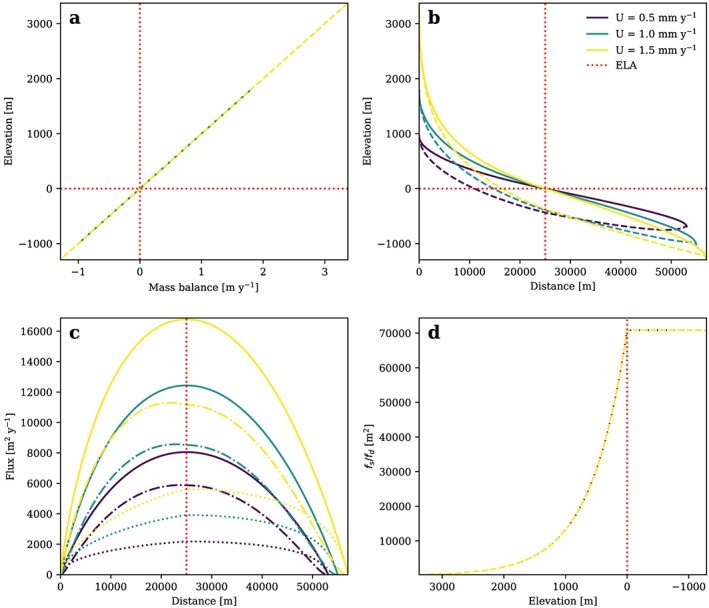
(a) Glacier mass balance, (b) ice surface (solid line) and bedrock surface (dashed line), (c) total flux (solid lines), flux from deformation (dash‐dotted lines), and flux from sliding (dotted lines), and (d) ratio of sliding parameter f_s_ and deformation parameter f_d_ as a function of elevation (δ = 0.006 K/m, ζ = 0.09) of a glacier longitudinal profile in topographic steady state plotted for l = 1 and different rock uplift rates. ELA = equilibrium line altitude.

The temperature dependencies of *f*_*d*_ and *f*_*s*_ lead to a strong increase in glacial relief as reduced basal sliding has to be compensated by steeper slopes (Figure 4). This causes an increase in ice flux and results in a longer glacier. The sliding parameter *f*_*s*_ asymptotically approaches zero sliding with increasing elevation which makes deformation more and more dominant (Figure 4d) as defined by equation (15).

## 3 Nonlinear Erosion Law

**Figure 5:**
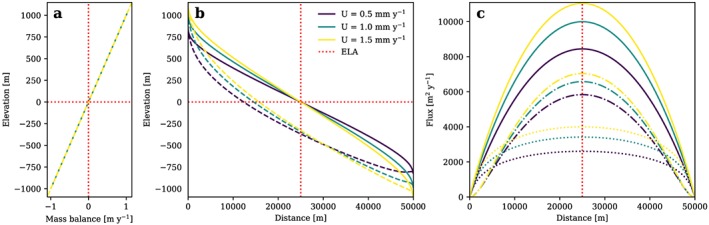
(a) Glacier mass balance, (b) ice surface (solid line) and bedrock surface (dashed line), and (c) total flux (solid lines), flux from deformation (dash‐dotted lines) and flux from sliding (dotted lines) of a glacier longitudinal profile in topographic steady state plotted for l = 2 and K_g_=1 × 10^−5^ m^−1^ year and different rock uplift rates. ELA = equilibrium line altitude.

Recent evidence suggests a nonlinear glacial erosion law (equation (2)) with a sliding velocity exponent of *l* = 2 (Herman et al., 2015; Koppes et al., 2015). Earlier constraints on the glacial erosion law from various field data suggest that *l* = 1 and *K*_*g*_≈1 × 10^−4^ derived from spatially integrated erosion rates and sliding velocities (Humphrey & Raymond, 1994; Koppes & Montgomery, 2009; Riihimaki et al., 2005). Due to the trade‐off between *K*_*g*_ and *l* these observations can also be explained with a nonlinear erosion law and an adjusted *K*_*g*_ (Herman et al., 2015). *l* only appears in equation (3) in our approach, and we can thus apply various sliding velocity exponents without the necessity of other adaptations. We perform the adjustment of *K*_*g*_ to *l* = 2 for the reference scenario of a simple glacier such that the resulting glacial steady state longitudinal profile with *l* = 2, *K*_*g*_=1 × 10^−5^ m^−1^ year and *U* = 1×10^−3^ m/year (Figure 5) is identical with the one presented in Figure 2 for *l* = 1, *K*_*g*_=1 × 10^−4^, and *U* = 1×10^−3^ m/year. Such a redefinition of *K*_*g*_ for a nonlinear erosion law can be done to fit a certain reference scenario, but a higher *l* reduces the sensitivity of glacial steady state profiles to external forcing. In contrast to the scenario with *l* = 1 (Figure 2), glacial relief, ice thickness and consequently ice flux are less sensitive to changes in rock uplift rate for a nonlinear erosion law (Figure 5).

## 4 Scaling Relations in a Glacial Topographic Steady State

A scaling relation describes how one variable depends on another and thus depicts the sensitivity of a variable to different kinds of system changes. Such scaling relations are fundamental properties of any system and have been established for the fluvial realm. They include, for example, the dependence of channel slope on drainage area (Flint, 1974; Hack, 1957) or the dependence of fluvial relief on rock uplift rate (Whipple & Tucker, 1999; Wobus et al., 2006). For glacial topography, however, such scaling relations are still missing for a coupled system including climate and tectonics. The approach presented above can be utilized to derive such scaling relations for a glacial equilibrium under the assumption that the ELA and the horizontal position of the equilibrium line are constant. We first report basic scaling for sliding‐ or deformation‐dominated versions of a simple glacier to constrain the fundamental controls that tectonics and climate exert on glacier shape. Subsequently, we analyze the influences of spatial variations in glacier mass balance gradient and ice temperature in a mixed scenario including both sliding and deformation which lead to more complicated scaling relations and nonuniform scaling exponents. In all analyses scaling relations for ice thickness *H* and ice surface slope d*h*/d*x* have been derived by applying our approach to different rock uplift rate and glacier mass balance scenarios and by artificially varying *l* and *n* to test the sensitivity of the relations to these exponents. Investigated rock uplift rates and glacier mass balance gradients were chosen to cover a large variety of field data and range from 0.01 to 10 mm/year and from 1 × 10^−4^ to 1 × 10^−2^, respectively. Spatially uniform *U* and *K*_*g*_ are assumed. We interpret the derived scaling relations in section 6.

### 4.1 Basic Scaling Relations

We explore basic scaling relations for a glacial topographic steady state for two simplified cases in which either sliding (*f*_*s*_≫*f*_*d*_) or deformation (*f*_*s*_≪*f*_*d*_) are assumed to be the dominant mechanism of ice motion in a simple glacier, which we define as temperate with constant temperature and mass balance gradient (Figure 2). Such a glacier has an ice flux that is symmetric about the ELA and ice thickness *H* and surface slope d*h*/d*x* depend on both rock uplift rate and glacier mass balance. Note that the scaling of ice surface slope is also valid for glacial relief as the horizontal position of the ELA is fixed in our approach and ice surface and bedrock elevation are identical at the glacier origin. The basic scaling regimes with *U* and *K*_*g*_ depend on the exponent of the erosion law *l* and the exponent of Glen's law *n* (Table 2). The scaling with the mass balance gradient *β*, the ice flux *q*, and the sliding and deformation parameters *f*_*s*_ and *f*_*d*_ is independent from the erosion law and only depends on *n*. The scaling with *q* also depends on *n* with the exception that it is always linear for *H* in a sliding‐dominated scenario where *H* is just given by *q*/*u*_*s*_.

**Table 2:** Exponents of H and dh/dx Scaling With the Governing Parameters of the Shallow Ice Approximation for Sliding‐Dominated (f_s_≫f_d_) and Deformation‐Dominated (f_s_≪f_d_) Scenarios

Scenario	$U_{f_{s}\gg f_{d}}$	$U_{f_{s}\ll f_{d}}$	$\beta_{f_{s}\gg f_{d}}$	$\beta_{f_{s}\ll f_{d}}$	$q_{f_{s}\gg f_{d}}$	$q_{f_{s}\ll f_{d}}$	*f*_*s*_	*f*_*d*_
*H*	$-\frac{n-1}{2\mathrm{nl}-l}$	$-\frac{n-1}{l(n^{2}+n)-l}$	$\frac{n}{2n-1}$	$\frac{n}{n^{2}+n-1}$	1	$\frac{1}{n}$	$-\frac{1}{2n-1}$	$-\frac{1}{n^{2}+n-1}$
$\frac{dh}{dx}$	$\frac{n}{2\mathrm{nl}-l}$	$\frac{2n-1}{l(n^{2}+n)-l}$	$-\frac{n-1}{2n-1}$	$-\frac{n-1}{n^{2}+n-1}$	$-\frac{n-1}{n}$	$-\frac{n-1}{n^{2}}$	$-\frac{1}{2n-1}$	$-\frac{n}{n^{2}+n-1}$

The scaling relations derived from our iterative model show that ice surface slope d*h*/d*x* increases with rock uplift rate and decreases with mass balance gradient, while ice thickness *H* behaves in the opposite way. Both variables thus represent the competition between tectonics and climate in shaping glacier steady state characteristics. The scaling relations are characterized by positive exponents <1 and negative exponents >−1, which indicates that the sensitivity to both tectonic and climatic forcing decreases when the magnitude of forcing increases. In both a sliding‐ and a deformation‐dominated regime steady state *H* and d*h*/d*x* are more sensitive to forcing in case of a linear erosion law than in case of a nonlinear law (Table 2), which is also depicted in the example profiles in Figures 2 and 5. *H* and d*h*/d*x* decrease with increasing *f*_*s*_ and *f*_*d*_. The scaling of glacier mass balance itself follows the scaling of relief and thus d*h*/d*x*.

Note that the scaling exponents for d*h*/d*x* and *H* derived from our model differ from those given by Headley et al. (2012). For example, Headley et al. (2012) report a linear relationship between *U* and d*h*/d*x* in a sliding‐dominated scenario with *n* = 3 and *l* = 1 and an exponent of 0.5 for *l* = 2, while the exponent derived here is 0.6 for *n* = 3 and *l* = 1, and 0.3 for *l* = 2. Similarly, the scaling exponent of *H* with *U* is −1 (*l* = 1) or −0.5 (*l* = 2) according to Headley et al. (2012), but −0.4 (*l* = 1) or −0.2 (*l* = 2) in this study. This is a consequence of considering the glacier mass balance as a function of elevation instead of distance along the glacier, which leads to a feedback between ice surface elevation and glacier mass balance.

### 4.2 Complicated Scaling Relations

**Figure 6:**
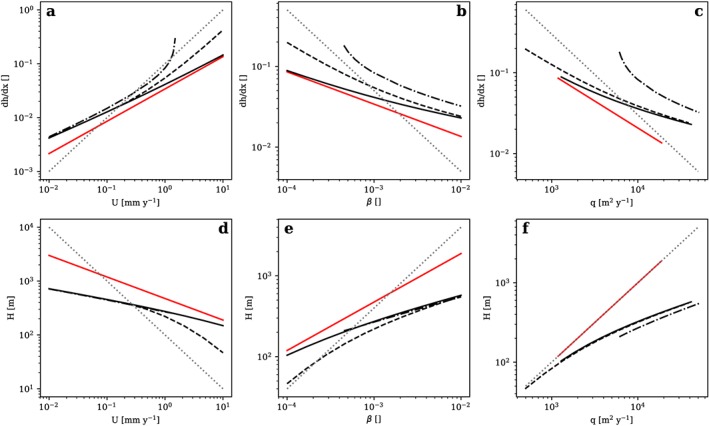
Scaling relations for dh/dx (a–c) and H (d–f) with U, β, and q, respectively. Sliding‐dominated reference scenario (solid red line, scaling exponents are 0.6 [a], −0.4 [b], −0.67 [c], −0.4 [d], 0.6 [e], and 1 [f]); power law scaling reference for orientation with exponent = 1 (a, e, and f) and exponent = −1 (b, c, and d; dotted gray line); sliding‐deformation mix (solid black line); sliding‐deformation mix with a glacier mass balance cutoff (dashed black line); sliding‐deformation mix with elevation‐dependent ice viscosity and basal sliding (dash‐dotted black line). All scaling regimes determined for the accumulation part of the glacier, for l = 1, n = 3. Mass balance cutoff B_m_ for β and q scaling relationships defined to depend linearly on β. Note the logarithmic scales: the slope of the graphs depicts the scaling exponents.

The basic relations between rock uplift rate, glacier mass balance, ice thickness, and ice surface slope for sliding‐ or deformation‐dominated glaciers all follow power laws. However, the scaling becomes complicated if ice sliding and deformation interact. Furthermore, scaling relations may be complicated by a break or cutoff in glacier mass balance and an elevation dependence of ice viscosity and basal sliding. Such scaling relations are difficult to report and interpret, but we suppose that a realistic scenario of a glacial equilibrium likely deviates from a simple glacier by incorporating a mix of ice deformation and sliding, a mass balance cutoff, or some temperature dependence. We thus report model implications on the related scaling of ice surface slope and ice thickness with rock uplift rate, mass balance gradient, and ice flux (Figure 6). Scaling exponents are not constant but vary with *U*, *K*_*g*_, *β*, and *q* in these scenarios.

For a scenario allowing the interaction of ice sliding and deformation, the exponent describing the relation between d*h*/d*x* and *U* increases with *U* (Figure 6a, solid black line), while the exponent describing the relation between d*h*/d*x* and *β* decreases with *β* (Figure 6b, solid black line). Similar scaling relations emerge with the other parameters (Figures 6c–6f, solid black lines). However, scaling exponents for d*h*/d*x* and *H* with *U*, *β*, and *q* again remain substantially <1 or >−1 (Figure 6, dotted gray lines for reference). Furthermore, the slopes of the graphs in Figure 6 are also mostly lower than in the sliding‐dominated scenario (Figure 6, solid red lines) which indicates a reduction of sensitivity to tectonic and climatic forcing.

In the case of a glacier mass balance cutoff, the scaling exponent of d*h*/d*x* with *U* substantially increases with *U* and approaches unity because, with increasing rock uplift rates, larger parts of the glacier are located above the mass balance cutoff elevation and hence become subject to uniform mass balance (Figure 6a, dashed line). In contrast, ice surface slope decreases at a reduced pace with increasing *β* and *q* (Figures 6b and 6c, dashed lines). The opposite behavior is observed for ice thickness *H* (Figures 6d–6f, dashed lines).

The consideration of temperature‐ and hence altitude‐dependent sliding and deformation parameters again changes the scaling behavior (Figure 6a, dash‐dotted line). In such a scenario, higher topography induced by higher rock uplift rates leads to lower air and ice temperatures and consequently reduced deformation and sliding parameters. This demands for steeper slopes to match the required sliding velocity which again increases mountain height (compare Figure 4). This positive feedback results in a very strong increase of d*h*/d*x* and thus glacial relief with *U*. This extreme development, however, is to some part owed to the simplicity of our model where ice temperatures equal air temperature and other processes such as periglacial rock wall retreat are not included to erode steep slopes. Nevertheless, our model implies scaling exponents ≥1 due to the feedback in such a scenario where glaciers approach a cold‐based state at high elevations.

*H* and d*h*/d*x* are generally less sensitive to changes in *U*, *β*, and *q* in a regime with a combination of sliding and deformation than in the sliding‐dominated reference scenario (Figure 6). Higher sensitivity of d*h*/d*x* and *H* in mixed scenarios is only observed in the case of temperature‐dependent ice movement, for high *U* and/or low *β* and *q*, and vice versa. Similar relationships can also be described for a nonlinear erosion law with *l* = 2. In this case, the sensitivity of ice surface slope and ice thickness to rock uplift rates is further reduced and the scaling exponents approximately halve, similar to the simplified scenarios described in Table 2. For example, if *l* = 2, the scaling exponents of d*h*/d*x* with *U* are generally ≤0.3, with exceptions for substantial relief above the mass balance cutoff and cold ice in high elevations.

## 5 Discontinuities and Valley Width

Our steady state model of glacial topography only allows predictions about glacier longitudinal profiles. Conclusions about the plan view shape of a steady state glacier or valley network cannot be derived. However, some additional variables can be prescribed to the 1‐D model to evaluate the possible effects of tributaries and changes in lithology or valley width.

### 5.1 Valley Steps

**Figure 7:**
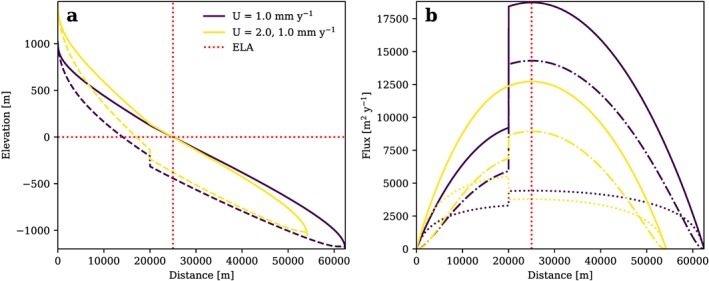
Effect of discontinuities such as a glacier confluence (purple) and a fault (yellow) on the shape of a glacial steady state longitudinal profile (a) and ice flux (b). Both discontinuities are located 5‐km up‐valley of the equilibrium line altitude. (ELA). In the case of the glacier confluence, q is assumed to double at the confluence, in the case of a fault rock, uplift rate doubles uphill of the fault.

The analyses presented so far imply that glacial steady state valleys have a smooth and gradually changing topography. Valley steps as observed along actual glacial valleys are often interpreted as transient features. However, in many cases distinct valley steps are a result of glacier confluences (e.g., Anderson et al., 2006; MacGregor et al., 2000), which have not been considered in our model so far. In addition, geological discontinuities marking differences in rock uplift rates and/or erodibility may cause abrupt changes in valley morphology and glacier characteristics (Headley et al., 2013). In Figure 7 we show model simulations that attempt to reproduce both situations.

In the first case, we set ice flux to double 5‐km up‐valley of the ELA, which represents the confluence of two similar ice streams. Figure 7a shows that, if such valley junctions exist in steady state, a distinct valley step would develop at the glacier confluence. At the confluence, the ice flux doubles (Figure 7b), but ice thickness only increases by about 1/3 due to scaling properties and a change in trade‐off between flux from sliding and deformation. The increase in ice thickness downstream of the convergence (i.e., 20‐km downstream from the top of the glacier) also leads to a slight decrease in bedrock and ice surface slopes.

An abrupt change in *U* or *K*_*g*_ along the glacial profile has an effect similar to a glacier confluence. In the case of a two‐fold decrease of rock uplift rate downstream of a fault (Figure 7), a similar but lower bedrock step develops accompanied by an increase of ice thickness and a clear decrease of bedrock and ice surface slopes. Note that in both scenarios, maximum ice thickness may be located below the ELA depending on the location of the glacier confluence or fault. In the first case, erosion rate and sliding velocity are uniform, while in the latter case ė and *u*_*s*_ vary as they have to match *U*. Headley et al. (2012) provide similar scenarios but without the feedback between glacier mass balance and ice surface elevation.

### 5.2 Accumulation Area Ratio and Valley Width

Glacial equilibrium profiles derived from uniform *U* and *K*_*g*_ and linear *B* (i.e., only linearly dependent on ice surface elevation) have an ice flux pattern that is symmetric about the ELA. A nonlinear glacier mass balance over elevation as introduced in section 2.2 leads to an asymmetric ice flux and hence to a deviation from equally sized accumulation and ablation areas. Such deviations also exist in real‐world glaciers with Ablation Area Ratios (AARs) generally >0.5 (e.g., Gross et al., 1977; Meier & Post, 1962; Porter, 1975). In contrast to our model, the AAR in real‐world glaciers does arise not only from differences in the 1‐D ice flux above and below the ELA but also from the hypsometry of the glacier, the distribution of glacier area over elevation. However, the shape and elevation of the steady state glacier profile are a priori unknown in our analysis and we thus adopt an approach of Anderson et al. (2006). We utilize a function that was introduced by Oerlemans (2008) to model glacier width, *w*, along its length based on observations of alpine glaciers with a wide accumulation basin and a narrow tongue. The width is given by

(16) $$w=w_{0}+w_{1}xe^{-\mathrm{kx}}$$

where *w*_0_ is the width at the terminus, *w*_1_ is a scaling parameter, and *k* is a shape parameter. The area of the glacier can be obtained by analytically integrating width along the glacier profile:

(17) $$A=w_{0}x+w_{1}(-e^{-\mathrm{kx}}k^{-2}(\mathrm{kx}+1))-C$$

with

(18) $$C=w_{0}x_{0}+w_{1}(-e^{-kx_{0}}k^{-2}(kx_{0}+1))$$

This allows two different approaches. Either all three parameters in equation (16) can be chosen to fit observations of glacier width or one parameter is left undefined and the system is solved for a width distribution that yields a predefined AAR at the ELA. To derive a glacier in topographic steady state with a predefined width along its length, we fix *k*, *w*_0_, and *w*_1_ as defined in Table 1. Our one‐dimensional model cannot be used to derive any information on equilibrium valley width, and thus, this addition is implemented solely to estimate its possible influence on steady state topography. Alternatively, other width formulations could be chosen (e.g., Anderson et al., 2006) or glacier width along with mass balance may be derived from a real‐world or modeled glacier that is thought to closely match an ice mass in topographic equilibrium.

**Figure 8:**
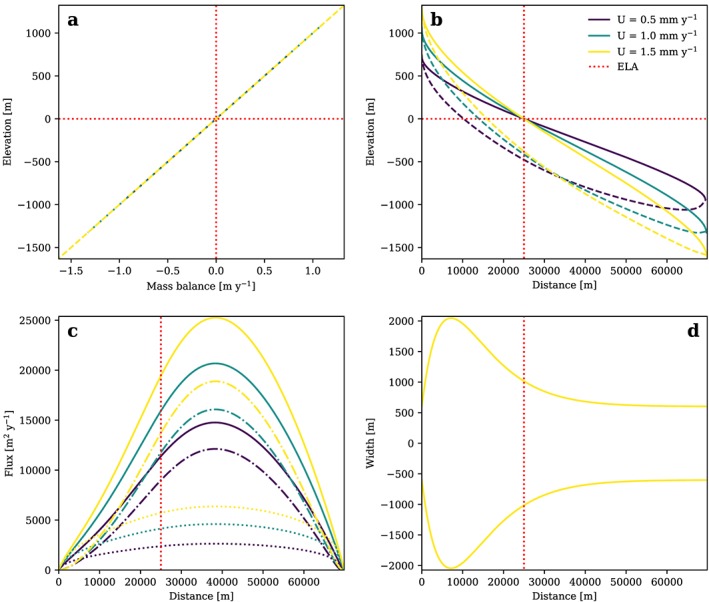
(a) Glacier mass balance, (b) ice surface (solid line) and bedrock surface (dashed line), and (c) total flux (solid lines), flux from deformation (dash‐dotted lines), and flux from sliding (dotted lines), and (d) width of a glacier longitudinal profile in topographic steady state plotted for an Ablation Area Ratio of 0.6, l = 1, different rock uplift rates, and a mass balance ratio γ of 1.2. Note that the width is assumed to be independent from both U and glacier mass balance. ELA = equilibrium line altitude.

The wide accumulation area in conjunction with the narrow tongue defined by our formulation of glacier width leads to channeled ice and an increased ice thickness below the ELA which results in an increased glacier length (Figure 8). In this steady state scenario maximum *H* and *q* are located below the ELA while glacial erosion and *u*_*s*_ are uniform.

The way glacial relief, ice surface and bedrock slopes, and ice flux are affected by glacier width of course depends strongly on the formulation. With a fixed width, the AAR changes with rock uplift rate in our approach, if influences like temperature‐dependent sliding or a mass balance cutoff are included. In contrast, with a fixed AAR, the width of the glacier has to change with the governing parameters *U*, *K*_*g*_, and *β*. It is difficult to argue whether this is a realistic scenario for a glacial steady state as the related hypsometry remains essentially unknown. In any case, absolute values of *H* and d*h*/d*x*, and thus glacier shape and topography, are affected by including glacier width and the AAR, but related scaling relations do not (fixed width) or only marginally (fixed AAR) depend on these influences for the tested parameter ranges. The only exception is a combination of a mass balance cutoff with a fixed AAR for *U* > 2×10^−3^ m/year.

## 6 Implications for Steady State Relief

The limiting effect of a climatically induced glacial buzzsaw on the height of mountain ranges has been extensively discussed in the literature (see section 1). The buzzsaw concept assumes that climate controls the amount of topography present above the ELA, while the rock uplift rate has little or even no relevance. This view is supported by analyses of hypsometric patterns in orogens worldwide and numerical landscape evolution models that show that glacial erosion modifies the hypsometry and reduces the overall relief of mountain landscapes (e.g., Brozović et al., 1997; Egholm et al., 2009; Mitchell & Montgomery, 2006). However, such models often do not incorporate tectonic uplift and can only simulate glacial erosion over a limited amount of time, typically one or several glacial cycles. Similarly, most glaciated mountain landscapes are likely to be in a transient state and related observations are thus only a snapshot of a system in an unknown evolutionary state. In contrast, constraints on steady state profiles and related glacial relief can be derived from our model, at least in 1‐D and under the assumption that the horizontal position of the ELA is constant. In such an analysis it seems adequate to consider scaling relationships of two indicators, glacial relief above the ELA and mean bedrock elevation, as both definitions have been used to describe the impact of cold climate on mountain topography. When predicting mean bedrock elevation in absolute values in a glacial topographic steady state, however, limitations arise from a 1‐D approach as the related hypsometry remains unknown. Nevertheless, we are still able to test whether or not steady state mean bedrock elevation above the ELA changes with rock uplift rate in our one‐dimensional approach. In any case, a discussion of the role of rock uplift rate in controlling glacial steady state relief and mean elevation seems to be most useful, if only to compare it to a nonglacial steady state scenario.

### 6.1 Comparison of Glacial and Fluvial Steady State Relief

In defining glacial steady state topography we were inspired by the work of fluvial geomorphologists who constrained the most important long‐term controls on fluvial topography and the shape of related steady state landscapes (Flint, 1974; Hack, 1957; Whipple & Tucker, 1999). In a fluvial steady state, erosion rate balances rock uplift rate ( U=ė), and thus,

(19) $$U=K_{f}D^{i}{\left(\frac{dz}{dx}\right)}^{j}$$

where *D* is drainage area and *i* and *j* are constants. Note that drainage area (*i*) and slope (*j*) exponents are commonly called *m* and *n* in related literature. The scaling of slope with *U* is then determined by the slope exponent *j* from

(20) $$\frac{dz}{dx}={\left(\frac{U}{K_{f}}\right)}^{1/j}D^{-i/j}$$

The value of *j* is still debated. In many modeling studies *j*≈1 is assumed (e.g., Perron & Royden, 2013; Wobus et al., 2006), while observations indicate *j* > 1, i.e., a nonlinear scaling of fluvial steady state relief with rock uplift rate, with a scaling exponent <1 (e.g., Kirby & Whipple, 2012; Lague, 2014; Scherler et al., 2017).

The scaling regimes for slope above the ELA in a glacial steady state presented in section 4 are derived from analyzing mean ice surface slope and are thus also valid for glacial relief above the ELA, as the horizontal position of the ELA is a fixed boundary condition in our approach and ice thickness approaches zero at the glacier origin, which makes ice surface and bedrock elevations identical. From this it becomes clear that rock uplift rate exerts some control on mountain height in glacial steady state landscapes for both linear and nonlinear erosion rules and leads to steepening of the ice and bedrock surfaces, which corresponds to findings of Brocklehurst and Whipple (2007); Pedersen et al. (2010). It is also clear that the dependence of glacier mass balance on altitude reduces the sensitivity of mountain height to rock uplift rate in a glacial steady state. This effect emerges from a feedback between mountain height and glacier mass balance. Higher rock uplift rates can be expected to increase mountain height in both fluvial and glacial landscapes, but in the latter, the increase is reduced due to a resulting increase in glacier mass balance and hence ice flux, sliding velocity and glacial erosion, which counteracts the growth of relief. The scaling exponent of d*h*/d*x* with *U* is ≤0.6 for temperate glaciers, if a linear erosion law (*l* = 1) is assumed (Figure 6a). This is similar to scaling exponents reported for the fluvial realm (Kirby & Whipple, 2012; Lague, 2014). However, recent evidence suggests a nonlinear glacial erosion law with *l* = 2 (Herman et al., 2015; Koppes et al., 2015). In this case, glacial scaling exponents approximately halve. The scaling exponent of d*h*/d*x* with *U* reduces to ≤0.3, which is substantially lower than observations on fluvial relief‐uplift scaling (Kirby & Whipple, 2012; Lague, 2014; Scherler et al., 2017). This comparison suggests that, while both fluvial relief and glacial relief above the ELA depend on rock uplift rate, glacial relief may be less sensitive to tectonic forcing under temperate conditions. Contrarily, if a different modeling approach is chosen, the width of a glacially‐dominated orogen seems to be more sensitive to tectonic and climatic forcing than the width of a fluvially dominated orogen (Tomkin & Roe, 2007).

The scaling of mean bedrock elevation above the ELA with *U* deviates from a power law even in the simplified scenarios with either sliding‐ or deformation‐dominated glaciers. This is a result of the influence of both d*h*/d*x* and *H* on the horizontal position of the bedrock‐ELA intersection. This leads to a variable scaling exponent for very low uplift rates where *H* becomes very large. However, for moderate to high rock uplift rates mean elevation above the ELA follows the scaling relations of relief and is thus also subject to a coupled control of tectonics and climate.

The sliding‐ or deformation‐dominated scaling regimes reported in Table 2 follow power laws and thus imply that, at least in these simplified cases, the increase in glacial relief above the ELA approaches zero and may thus become negligible for very high uplift rates. However, we have used empirically defined parameters for deriving our scaling regimes and find that uplift rates sufficiently high to approach a relief independent of tectonic forcing are orders of magnitudes higher than the highest reported long‐term rates (e.g., Enkelmann et al., 2011; Herman et al., 2013; Koppes & Montgomery, 2009; Lundberg & Dorsey, 1990; Tippett & Kamp, 1993). Furthermore, the presence of a mass balance cutoff due to the depletion of moisture with altitude and/or the reduced sliding properties of cold ice may lead to scaling exponents for d*h*/d*x* with *U* close to 1 (*l* = 1) or 0.5 (*l* = 2), or even higher (Figure 6). The importance of these two effects can be expected to increase with elevation and thus rock uplift rates and may hence oppose a possible reduction of relief sensitivity toward zero at very high rock uplift rates. This may contribute to explaining the presence of high relief in high latitudes such as the Denali Range, Alaska, where temperatures are generally lower than in temperate mountain ranges.

In addition to the metrics discussed above, the shape of the glacier longitudinal profile itself depends on *U*, *K*_*g*_, and *β*. The ice surface takes an S‐shape, concave in the upper, straight in the middle, and convex in the lower part. This S‐shape becomes more distinct with increasing *U* and/or decreasing *β*. Similarly, the concavity of the glacier bed is most pronounced for low *U* and/or high *β* with overdeepening close to the glacier terminus. With increasing *U* and/or decreasing *β* the bedrock profile straightens and eventually becomes S‐shaped as well. This applies to all investigated scenarios (Figures 2, 3, 4) and implies that glacial overdeepening and related phenomena such as glacial lakes and abundant valley infill are most pronounced in low‐uplift/high precipitation areas. However, different effects such as glacier length variations, valley confluences, localized basal water pressure or erodibility contrasts have been proposed to cause glacial overdeepening at different locations along a glacier profile (e.g., MacGregor et al., 2000, and references therein). Some of these mechanisms cannot be reproduced by our model or only occur in transient conditions.

Our model based on the SIA implies that ice thickness, glacial relief, and mean elevation above the ELA depend on rock uplift rates in glacial steady state longitudinal profiles. This opposes a purely climatic control on mountain height. However, in contrast to most fluvial regimes, the incorporation of glacier mass balance as an altitude‐dependent variable reduces the sensitivity of glacial relief to rock uplift rate. The reduction of this sensitivity depends on the glacier type, sliding and erosion laws and sliding and deformation properties.

### 6.2 Periglacial Erosion

Our study does not take into account the role of hillslope processes but solely reports the impact of glacial erosion on steady state topography based on simple theoretical formulations. Processes that denude permafrost‐affected hillslopes and rock walls undoubtedly play a major role in relief development of high‐mountain landscapes and recent studies have suggested the existence of a *periglacial buzzsaw* (Hales & Roering, 2005; Scherler, 2014), have explicitly included periglacial erosion in glacial landscape evolution models (Egholm et al., 2015; MacGregor et al., 2009) and have reported possible feedbacks between hillslopes and glaciers (Scherler et al., 2011; Ward et al., 2012). Nevertheless, similar to river incision controlling hillslope processes in the fluvial realm, glaciers represent the backbone of cold‐climate mountain landscapes and hence can be expected to exert a major control on relief development. Our analyses thus have the potential to serve as a benchmark for understanding the evolution of glacial mountain landscapes.

### 6.3 Climate Variations During the Quaternary

The direct application of our theoretical model to the real world is mainly challenged by the assumption of an ELA that is constant in space and time. The Quaternary climate was subject to substantial variation in ELAs and glacier extents over different time spans (e.g., Lisiecki & Raymo, 2007; Petit et al., 1999; Zachos et al., 2001). The mean climate of the Quaternary was likely a glacial one, although most probably not a full Last Glacial Maximum one (e.g. Kirkbride & Matthews, 1997; Porter, 1975). This raises the question of which average climate glacial landscapes would adjust to, in particular as different parts of the landscape were occupied by glaciers over different time spans (Anderson et al., 2006, 2012), and ice sliding conditions may have changed with climate. Different parts of the landscape may have adjusted to different average climate conditions, and fluvial processes may have altered or even fully transformed glacial terrain into a fluvial state during interglacial periods (Prasicek et al., 2015). On the other hand, glacial landscapes may asymptotically approach a steady state with rapid transformation in early stages (e.g., Leith et al., 2014; Pedersen et al., 2010), indicated in low‐lying trunk valleys where glacial erosion seems to have caused massive overdeepening and hanging tributaries only during glacial maxima (e.g., Jaboyedoff & Derron, 2005; Pomper et al., 2017).

Testing these influences on the state of glacial topography is beyond the scope of this theoretical study. However, most of the points raised above are not yet satisfactorily understood, which demonstrates the need for additional theoretical models and empirical studies. Although large parts of the present‐day glacial topography worldwide are most likely in a transient state, fully glaciated landscapes experiencing tectonic uplift may allow a comparison to steady state configurations. For example, Pedersen et al. (2010) suggest that in part of the Andes, the Himalaya and the North American Coastal Ranges erosion above the ELA may be in equilibrium with uplift rates. Beyond aiming to detect glacial steady state topography in the real world, spatial variations in the degree of similarity with a steady state topography may help to improve our understanding of landscape dynamics during the Quaternary.

## 7 Conclusions

We modeled the shape of glacial steady state longitudinal profiles in a coupled system in which the interaction of climate and tectonics controls glacial topography and ice thickness, based on the formulations of the shallow ice approximation and a glacial erosion law. We derived scaling relations for ice thickness and ice surface slope for sliding‐ and deformation‐controlled scenarios, as well as scenarios with a sliding‐deformation mix, and other influences such as a mass balance cutoff and temperature‐dependent ice viscosity and basal sliding properties. From these analyses we constrained the dependence of glacial relief and mean elevation on rock uplift rate and compared it to mathematical formulations of fluvial topography. Our modeling results imply that

1. both tectonics and climate control steady state ice thickness, ice surface slope, and glacial relief above the ELA;
2. ice surface slope, glacial relief, and mean elevation increase with rock uplift rate in a glacial steady state, with characteristic scaling exponents ≤0.6 in case of a linear glacial erosion law (*l* = 1) or ≤0.3 in case of a nonlinear glacial erosion law (*l* = 2);
3. ice surface slope, glacial relief, and mean elevation decrease with glacier mass balance gradient with characteristic scaling exponents ≥−0.4;
4. ice thickness decreases with rock uplift rate with exponents ≥−0.4 in case of a linear glacial erosion law (*l* = 1) or ≥−0.2 in case of a non‐linear glacial erosion law (*l* = 2);
5. ice thickness increases with glacier mass balance gradient with exponents ≤0.6;
6. substantially higher scaling exponents of ice surface slope, glacial relief, and mean elevation with rock uplift rate are possible in case of a low glacier mass balance cutoff or reduced sliding due to cold ice;
7. an increase in rock uplift rate raises mountain height in both fluvial and glacial steady state landscapes, but in the glacial realm glacier mass balance and hence ice flux increases with elevation, which counteracts the growth of relief and reduces the sensitivity to tectonic forcing;
8. a glacial buzzsaw concept that conforms to the shallow ice approximation and glacial erosion rules should incorporate the coupled impact of tectonic and climatic forcing on glacial steady state topography and the resulting reduction of sensitivity to rock uplift rates in comparison to a fluvial equilibrium.

We elaborated a quantitative relationship between glacial landscape characteristics and tectonic and climatic controls under the assumption of topographic steady state. Further research will focus on whether glacial landscapes that correspond to our steady state solution exist on our planet and to which average climatic conditions various glaciated mountain belts under different tectonic settings may have adjusted to.
